# Oxidative Stress-Inducing Anticancer Therapies: Taking a Closer Look at Their Immunomodulating Effects

**DOI:** 10.3390/antiox9121188

**Published:** 2020-11-27

**Authors:** Jinthe Van Loenhout, Marc Peeters, Annemie Bogaerts, Evelien Smits, Christophe Deben

**Affiliations:** 1 Center for Oncological Research (CORE), Integrated Personalized and Precision Oncology Network (IPPON), University of Antwerp, 2610 Wilrijk, Belgium; Marc.Peeters@uza.be (M.P.); evelien.smits@uza.be (E.S.); christophe.deben@uantwerpen.be (C.D.); 2 Department of Oncology, Multidisciplinary Oncological Center Antwerp, Antwerp University Hospital, 2650 Edegem, Belgium; 3 Plasma Lab for Applications in Sustainability and Medicine ANTwerp (PLASMANT), University of Antwerp, 2610 Wilrijk, Belgium; annemie.bogaerts@uantwerpen.be

**Keywords:** cancer therapy, oxidative stress, immune system

## Abstract

Cancer cells are characterized by higher levels of reactive oxygen species (ROS) compared to normal cells as a result of an imbalance between oxidants and antioxidants. However, cancer cells maintain their redox balance due to their high antioxidant capacity. Recently, a high level of oxidative stress is considered a novel target for anticancer therapy. This can be induced by increasing exogenous ROS and/or inhibiting the endogenous protective antioxidant system. Additionally, the immune system has been shown to be a significant ally in the fight against cancer. Since ROS levels are important to modulate the antitumor immune response, it is essential to consider the effects of oxidative stress-inducing treatments on this response. In this review, we provide an overview of the mechanistic cellular responses of cancer cells towards exogenous and endogenous ROS-inducing treatments, as well as the indirect and direct antitumoral immune effects, which can be both immunostimulatory and/or immunosuppressive. For future perspectives, there is a clear need for comprehensive investigations of different oxidative stress-inducing treatment strategies and their specific immunomodulating effects, since the effects cannot be generalized over different treatment modalities. It is essential to elucidate all these underlying immune effects to make oxidative stress-inducing treatments effective anticancer therapy.

## 1. Introduction 

Reactive oxygen species (ROS) is a collective term referring to unstable, reactive, partially reduced oxygen derivatives that are produced during metabolic processes within the mitochondria, peroxisomes and the endoplasmic reticulum (ER). A subset of ROS is also continuously generated by enzymatic reactions involving cyclooxygenases, nicotinamide adenine dinucleotide phosphate (NADPH) oxidases (NOX), xanthine oxidases, lipogenesis and through the iron-catalyzed Fenton reaction [1]. Examples of ROS include hydrogen peroxide (H_2_O_2_), superoxide anion (O_2_^•−^), singlet oxygen (^1^O_2_) and hydroxyl radical (^•^OH) [2]. Tight regulation of these ROS levels is crucial for cellular life. Therefore, cells benefit from a complex scavenging system based on different antioxidants, including superoxide dismutase (SOD), glutathione (GSH) peroxidase, peroxiredoxin, thioredoxin (Trx) and catalase [1]. Additionally to the strong antioxidant activity of the beforementioned enzymes, various non-enzymatic small-molecule antioxidants such as glutathione, ascorbic acid, vitamin E, polyphenolic compounds also act as scavengers for different types of ROS [3]. 

Cancer cells are characterized by increased production of ROS compared to normal cells. The persistently high levels of ROS can be explained by the imbalance between oxidants and antioxidants in cancer cells and the ongoing aerobic glycolysis by pyruvate oxidation in the mitochondria, also known as the Warburg-effect [4]. This is a consequence of hypoxia in the tumor microenvironment (TME) resulting from an imbalance between oxygen supply and consumption due to uncontrollable cell proliferation, altered metabolism and abnormal tumor blood vessel growth [5]. Cancer cells evolved mechanisms to protect themselves from this intrinsic oxidative stress and developed an adaptation mechanism by upregulation of pro-survival molecules and their antioxidant defense system to maintain the redox balance [6]. For instance, nuclear factor erythroid 2-related factor 2 (Nrf2), which is a transcription factor in the first line of antioxidant defense against oxidative stress, is often upregulated in cancer cells and supports cancer cell proliferation [7]. 

A low to moderate increase in intracellular ROS levels may result in activation of oncogenes (such as Akt), which are involved in cell proliferation, and inactivation of tumor suppressor genes, angiogenesis and mitochondrial dysfunction, thereby serving as a signaling molecule in cancer survival [4]. Conversely, when the levels of ROS are further elevated, they can overcome the defensive antioxidant system of cancer cells, causing cell death [8]. 

Consequently, there are two different approaches based on the redox balance to counteract cancer cells. In the first approach, oxidative stress can be decreased via scavenging intracellular ROS. For example, increasing intake of antioxidants (e.g., vitamin C and E) can deplete oxidative stress, subsequently causing growth inhibition and increased susceptibility to cell death in cancer cells, due to a crisis in energy production [9]. However, this antioxidant supplementation remains controversial [10]. Increasing evidence has shown that antioxidant supplementation fails to provide cancer protection and can even affect cancer mortality [11,12,13]. These observations are further supported and rationalized by recent studies demonstrating that oxidative stress can inhibit cancer progression and metastasis and that the GSH and Trx antioxidant systems, which are under the transcriptional regulation of Nrf2, may promote tumorigenesis and resistance to therapy [14]. 

The second approach is by increasing ROS levels in cancer cells and thereby crossing the threshold of cancer cell death. This can be done either by direct production of ROS via exogenous approaches or indirectly by increasing intracellular ROS concentrations via targeted inhibition of previously mentioned endogenous antioxidant systems in cancer cells. Several investigations are suggestive of the fact that the underlying mechanism of action and efficacy of conventional therapies (e.g., radiotherapy and chemotherapy) inducing cancer cell death, is the generation of elevated ROS levels during treatment [15,16,17].

In this review, we will focus on therapies related to this second approach and how they influence the TME, more specifically the immune cell compartment, to provide an overview of the effect of ROS induction on the antitumor immune response. 

### 1.1. Exogenous ROS Generation 

**Figure 1:**
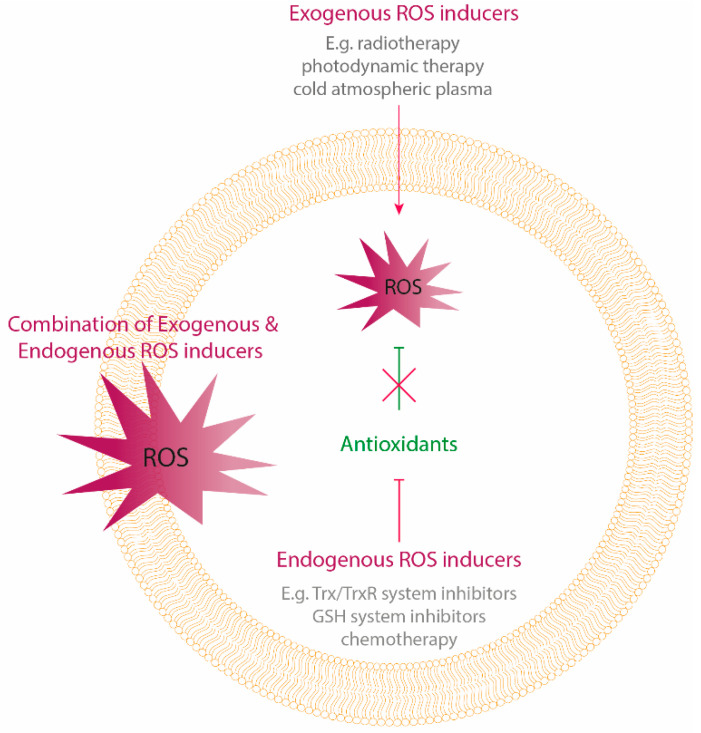
Oxidative stress-inducing treatment strategies. Oxidative stress can be induced by exogenous delivery of reactive oygen species (ROS) using physical treatment modalities, as well as by targeting the endogenous antioxidant system causing an intracellular accumulation of ROS. Cancer cells counteract exogenous delivery of high ROS levels by enhancing their antioxidant capacity. Therefore, a combination of both exogenous and endogenous ROS delivery by targeting the antioxidants, can be a promising anticancer strategy.

One mechanism of enhancing oxidative stress levels to target cancer cells is via exogenous delivery of ROS using different physical modalities (Figure 1). 

Ionizing radiation is widely used to treat many types of cancer. During radiation, cancer cells are eradicated through free radicals such as superoxide and hydroxyl radicals which are generated by radiolysis of water in extracellular environments and indirectly damage critical targets, such as DNA [15]. In addition, radiotherapy can also alter mitochondrial membrane permeability and activate NADPH oxidase, which in turn further stimulates ROS production [18]. Besides radiotherapy, other physical modalities that can induce a substantial increase in ROS levels are being investigated in cancer research, including photodynamic therapy (PDT) and cold atmospheric plasma (CAP) [19,20,21,22]. PDT is a light-based oncological intervention. Here, a photosensitizer is applied and subsequently activated by light. Upon activation, exogenously produced ROS is generated [23]. CAP is an ionized gas that can be produced at atmospheric pressure near room temperature. It is composed of reactive oxygen and nitrogen species, excited molecules, ions, electrons and other physical factors, such as electromagnetic fields and ultraviolet radiation [24]. 

### 1.2. Endogenous ROS Generation

The second mechanism of enhancing oxidative stress levels is via intracellular ROS accumulation through chemotherapy or targeted inhibition of the elevated antioxidant system (Figure 1). A lot of chemotherapeutic agents enhance intracellular levels of ROS and can alter the redox homeostasis of cancer cells. This amplification of ROS levels towards cytotoxic levels is one of the proposed mechanisms by which multiple chemotherapeutics induce tumor regression. The level of ROS generation is different among several compounds. Agents that generate high levels of ROS include anthracyclines (e.g., doxorubicin), platinum coordination complexes (e.g., cisplatin), alkylating agents (e.g., cyclophosphamide), camptothecins, arsenic agents and topoisomerase inhibitors, while nucleoside, nucleotide analogs, antifolates, taxanes and vinca alkaloids only generate low levels of ROS [25]. 

There are two mechanisms for elevated ROS production during chemotherapy, namely through mitochondrial ROS generation and inhibition of the cellular antioxidant system and thereby interfering with ROS metabolism in cancer cells [25]. Several agents, including arsenic trioxide, doxorubicin and cisplatin, have been reported to induce a loss of mitochondrial membrane potential and to inhibit respiratory complexes, leading to the disruption of mitochondrial electron transport chain (ECT) and electron leakage, which is a major source of elevated ROS levels [26,27,28]. 

The other mechanism for intracellular ROS accumulation is the inhibition of the antioxidant system during chemotherapy. For instance, imexon, a small-molecule used to treat advanced cancer of the breast, lung or prostate, binds to thiols such as GSH, causing a depletion of cellular GSH and consequently an accumulation of oxidative stress in cancer cells [29]. For some chemotherapeutics, more than one target site for ROS generation in cancer cells has been identified. For example, in addition to mitochondrial respiration, NADPH oxidase and thioredoxin reductase (TrxR) are other targets of arsenic trioxide induced oxidative stress, inducing apoptosis [30,31,32]. 

Besides chemotherapy, selective inhibitors that block components of the cellular antioxidant system are being studied as antitumor agents that enhance endogenous ROS production. For instance, depletion of the GSH antioxidant system can also be achieved by targeting its synthesis through buthionine sulfoximine (BSO), which has been shown to exhibit anticancer activities in various types of cancer. Furthermore, inhibitors of the Xc-cystine/glutamate antiporter (e.g., sulfasalazine) may also cause GSH depletion by inhibiting the uptake of cystine, the precursor of cysteine, which is a substrate for GSH synthesis [33]. Another antioxidant is the thioredoxin/thioredoxin reductase (Trx/TrxR) system, which is shown to be upregulated in cancer cells and is correlated with cancer aggressiveness and drug resistance [30]. TrxR is required to convert oxidized Trx into its functional reductive form, which can scavenge ROS [34]. TrxR activity can effectively be blocked by the gold compound auranofin that is clinically used as an antirheumatic drug and functions as a thioredoxin inhibitor. In different cancer cells, it has been preclinically shown to induce ROS-mediated cell death, since the ROS scavenger N-acetylcysteine prevents this cytotoxic effect [35,36]. This has led to the use of auranofin in several clinical trials involving non-small cell lung and ovarian cancer (NCT01737502 and NCT03456700). The small-molecule PX-12 is another example of an antioxidant inhibitor, since it inhibits Trx and is being used as therapy for advanced cancers in clinical trials [37,38]. 

### 1.3. Molecular Pathways Involved in Oxidative Stress-Inducing Therapies

**Figure 2:**
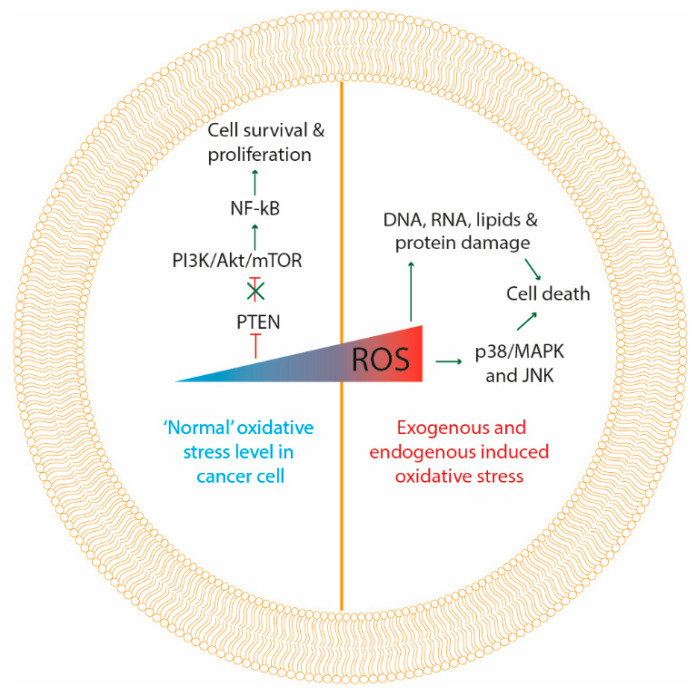
Molecular responses to oxidative stress. At moderate “normal” levels of oxidative stress in cancer cells (left side of the figure) ROS inactivate phosphatase and tensin homolog (PTEN) and unlock PI3K/Akt/mTOR pathway, which in turn activates NF-kB, consequently activating cancer cell survival and proliferation signaling. At high levels of oxidative stress induced by therapy, damage is produced to DNA, RNA, proteins, lipids and mitochondria, initiating apoptotic cell death. Additionally, high levels of ROS can activate p38/MAPK and JNK apoptotic signaling proteins, inducing cancer cell death.

Whether ROS augment tumorigenesis or lead to apoptosis, critically depends on the intracellular ROS levels. At moderate concentration, ROS inactivate phosphatase and tensin homolog (PTEN) and unlock the PI3K-depedent recruitment of its downstream kinases, such as Akt, which will, in turn, activate NF-kB, subsequently activating the cancer cell survival signaling cascade [39]. For instance, hydrogen peroxide can reversibly oxidize cysteine thiol groups of PTEN, causing the loss of their activity and promoting the activation of the PI3K/Akt/mTOR survival pathway, consequently leading to tumor cell survival [4]. Abundant high concentrations of ROS originating from exogenous and endogenous sources, produce oxidative damage to the DNA, RNA, proteins, lipids and mitochondria, initiating apoptotic cell death (Figure 2) [39,40]. 

In line with this, it has been shown that the cellular response to exogenous sources of ROS strongly varies with the intensity of the treatment [41,42,43]. For example, low dosages of PDT and radiotherapy have been shown to transiently activate several kinases and NF-kB involved in survival signaling [43,44]. In these non-toxic dosages of PDT, kinases that are important to initiate autophagy were shown to be activated [44]. Higher dosages of radiation and PDT activate the mitochondrial apoptotic pathway and additionally can also produce a sustained activation of MAPK families including p38, MAPK, ERK1/2 and JNK apoptotic signaling proteins (Figure 2) [40,45,46]. 

Inhibition of the antioxidant system of cells could also induce apoptosis of cancer cells. Trx is a physiological inhibitor of ASK1 located upstream of the p38/MAPK pathway, and therefore disrupts the p38/MAPK dependent apoptosis. As such, an inhibitor of the Trx/TrxR system could induce apoptosis due to the phosphorylation of p38/MAPK, as well as the activation of JNK and ERK [34]. Additionally, several studies have shown that Trx/TrxR inhibitors downregulate the PI3K/Akt/mTOR survival pathway, causing apoptosis of different types of cancer cells [34,47,48,49]. The same effect was observed using an inhibitor of the GSH antioxidant pathway [50]. 

### 1.4. Combinations of Different Oxidative Stress-Inducing Therapies 

It has been shown that radiotherapy, PDT, as well as other ROS-inducing therapies, could induce acquired resistance to therapy. Here, NF-kB is considered to be a key component in the rise of therapy-resistant cancer [43,51]. Suppression of the NF-kB activation pathway sensitized cells to radiotherapy-induced apoptosis by increasing activation of the JNK pathway [52]. Furthermore, it has been suggested that resistance to therapies that induce intracellular ROS production, such as chemotherapy (e.g., paclitaxel and doxorubicin) and radiotherapy, is correlated with an increased antioxidant capacity of cancer cells. Here, upregulation of Nrf2 after oxidative stress contributes to the therapy resistance in cancer cells [53,54]. Due to this complexity of redox homeostasis and adaptation-mediate resistance in tumor cells, ROS-inducing treatments may not always lead to an effective antitumor effect. To overcome resistance induced by oxidative stress and to maximally exploit the ROS-mediated cell death mechanism as a therapeutic strategy, it would be beneficial to combine therapeutic strategies that exogenously induce ROS together with compounds that suppress the cellular antioxidant system.

In several preclinical studies, inhibition of GSH or Trx antioxidant systems, downstream of Nrf2 signaling, has been demonstrated to sensitize different types of tumor cells towards radiotherapy [55,56,57]. BSO used to inhibit the GSH production, has been shown to sensitize lung, renal and head and neck cancer to radiation. The combination of radiation and GSH depletion by BSO resulted in the activation of the JNK signaling pathway, which resulted in triggering the intrinsic apoptotic pathway [55]. A combination of other agents to disrupt endogenous redox homeostasis was also proven to improve therapeutic efficiency and overcome tumor resistance to PDT [58,59]. However, in combination with BSO, a synergistic effect with PDT was only seen when BSO alone had negligible cytotoxicity. This indicates that cancer cells with intracellular high levels of antioxidants (e.g., GSH) will be more intrinsically resistant toward antioxidant inhibitors or radiation alone, but will effectively induce cell death when these exogenous and endogenous ROS inducers are combined [59,60,61]. Similar effects were seen when combining BSO with a platinum-based chemotherapy-inducing ROS [62]. Additionally, inhibitors of the Trx/TrxR system (such as PX-12, auranofin and motexafin gadolinium) have shown similar effects to enhance the response against exogenously therapy-induced ROS [63,64].

Hypoxia is also one of the most important causes of exogenous oxidative stress-inducing therapy failure, because of the shortage of ROS substrate oxygen. However, it is demonstrated that more ROS is produced in hypoxic conditions compared to non-hypoxic conditions. Although the specific mechanism has not been described, it appears that the source of the increased ROS levels generated under hypoxia is the mitochondria. Hypoxia increases ROS via the transfer of electrons from ubisemiquinone to molecular oxygen at the Qo sites of complex III of the mitochondrial electron transport chain [65]. Besides mitochondria, nitric oxide synthases (NOS) and NOX have also been implicated to increase ROS production during hypoxia [66]. Moreover, NO and its derivates are a specific group of ROS synthesized by NOS. Since the inducible NOS (iNOS) is a hypoxia response gene, the generation of NO is significantly increased in tumor cells under hypoxic conditions [67]. As such, hypoxic tumor cells heavily rely on the antioxidant defense system to maintain ROS balance, making them vulnerable to inhibition of this antioxidant system [66]. For instance, BSO produces a more pronounced GSH depletion in regions of hypoxia, since GSH levels are higher in hypoxic compared to non-hypoxic regions [68]. Furthermore, auranofin was able to overcome hypoxic radiation resistance and the effect could be further amplified by combining auranofin with BSO, leading to significant tumor growth delay and increased survival rate of tumor-bearing mice [56,57]. Therefore, inhibition of the antioxidant system could be effective to counteract hypoxia-induced therapy resistance [69]. 

Since the upregulation of NF-kB is also a key player in acquired resistance to ROS-inducing therapy, inhibition of this transcription factor could enhance the anticancer effect. For example, auranofin has shown to decrease the expression of NF-kB, thereby overcoming acquired therapy resistance [70,71]. Similar effects were obtained when inhibiting GSH metabolism [72]. However, it should be mentioned that the activation of NF-kB is also responsible for inflammatory responses, which can induce cross-presentation of tumor antigens and stimulate antitumor immune responses [73]. This indicates that it is important to take into account the effects of exogenous and endogenous ROS-inducing therapies on the antitumoral immune system. 

## 2. Indirect and Direct Effects of Oxidative Stress-Inducing Therapies on the Antitumoral Immune Response

**Figure 3:**
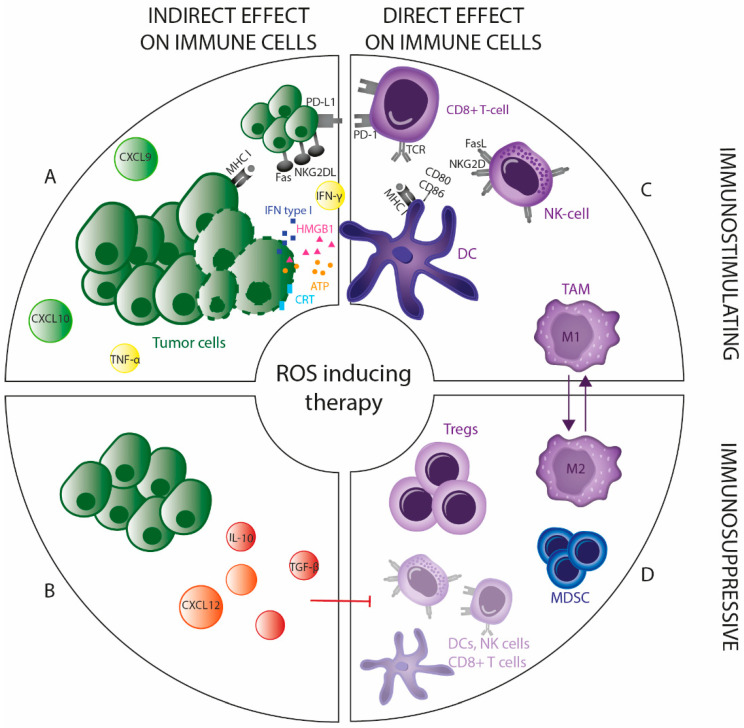
Direct and indirect immunomodulating effects of ROS-inducing therapy. The immunomodulating effects after ROS-inducing therapy can be divided into direct and indirect effects being immunostimulatory and/or immunosuppressive. (**A**) ROS-inducing therapy triggers recruitment and activation of DC by inducing immunogenic tumor cell death (ICD). Additionally, treated tumor cells can secrete cytokines (e.g., IFN-γ and TNF-α) and chemokines (e.g., CXCL10 and CXCL9), and can modulate their surface molecules (e.g., MHC-I, PD-L1 and NKG2DL), thereby increasing their susceptibility to T cell and NK cell-mediated cytotoxicity. (**B**) Immunosuppressive cytokines (e.g., IL10 and TGF-β) and chemokines (e.g., CXCL12) can also be secreted by tumor cells treated with ROS inducers, suppressing immunostimulatory immune cells (DCs, T cells and NK cells) and promoting immunosuppressive immune cells (Tregs and MDSCs). (**C**) Depending on the intensity, ROS-inducing treatment skews TAMs towards a more antitumoral (M1) or protumoral (M2) phenotype. (**D**) T cells and NK cells are sensitive to oxidative stress-inducing treatments, compared to Tregs which are more resistant to these toxic effects.

In recent years, it has become clear that the immune system is a strong ally in the fight against cancer. ROS-inducing treatments have significant effects on the immune system, which can be either immunostimulatory or, in some circumstances, immunosuppressive [74,75]. Here we will discuss the direct and indirect effects of these therapies on the immune system, which are either immunostimulatory or immunosuppressive (Figure 3, Table 1). 

### 2.1. Indirect Effects on the Antitumoral Immune Response 

#### 2.1.1. Priming of an Adaptive Immune Response 

Tumor cells undergoing cell death in response to oxidative stress-induced therapy have the capacity to trigger an adaptive anticancer immune response, a concept known as immunogenic cell death (ICD). This is a unique type of cell death characterized by the release of danger signals after treatment of tumor cells, leading to the effective presentation of tumor antigens and subsequent priming of antigen-specific T cells. This process enhances the elimination of tumor cells and generates immune memory against the tumor antigens, thereby reducing the chance of recurrence [124]. Mechanistically, ICD induction requires ROS generation and further ROS-based ER stress [125]. In literature, there are already comprehensive reviews and research articles that discuss physical ROS-inducing modalities, such as radiotherapy, PDT and CAP, which have been shown to elicit effective antitumor immunity [76,77,78,79,80]. Additionally, chemotherapeutics which have been proven to be ICD inducers (e.g., oxaliplatin and doxorubicin) are accompanied by ROS-induced cytotoxicity [81]. 

Danger signals released during ICD include the release of adenosine triphosphate (ATP), which attracts dendritic cells (DCs) into the tumor and can stimulate the release of interleukin (IL)-1β, which promotes T cell priming. Moreover, calreticulin is expressed on the surface of the treated tumor cells, which promotes phagocytosis of these cells by DCs. ICD is also associated with high-mobility group box 1 (HMGB1) release, which facilitates antigen presentation and type-I interferon (IFN) secretion, mediating DC maturation [78,82,83]. Release of ATP, however, also modulates immunosuppressive properties of myeloid-derived suppressor cells (MDSCs) and can contribute to tumor growth and inhibition of antitumor immunity [110]. 

#### 2.1.2. Recruitment of Leukocytes 

Low infiltration of effector T cells and other leukocytes (e.g., NK cells) into the tumor represents a major obstacle for cancer immunotherapy [126]. Here, therapy can facilitate leukocyte infiltration by generating chemoattractants to induce leukocyte extravasation.

The most relevant signals regulating leukocyte infiltration are therapy-induced chemokines secreted by treated tumor cells and/or stromal components. For instance, several exogenous ROS inducers (e.g., radiotherapy, PDT and CAP) induce CXCL9 and CXCL10 secretion, which attracts T cells and thereby enhances tumor control [83,84,85,86]. By contrast, CXCL12 induced by radiotherapy can attract tumor-promoting MDSCs [111,112]. This underscores the double-edged sword of oxidative stress-induced therapy in the antitumor immune response. Additionally, a high dose of platinum-based chemotherapy is considered immunosuppressive, causing lymphopenia and neutropenia. However, complementary to other ROS-inducing treatments, it has been shown that low dose treatment enhances the T cell response with an increased number of T cells due to secreted chemokines (CXCL9, CXCL10 and CCL5) after treatment [87]. Other chemotherapy-based treatments are also able to upregulate the expression of chemokines receptors ligands in the TME, subsequently enhancing T cell recruitment [88,89,90]. 

#### 2.1.3. Modification of the Related Surface Molecules 

Susceptibility of tumor cells to T cell and NK cell-mediated cytotoxicity can be modulated by the expression pattern of surface molecules, including major histocompatibility complex (MHC)-I, MHC-II, NK cell ligands, costimulatory receptors and death receptors. The MHC class I is vital for the presentation of endogenous and potentially tumor-specific antigens to cytotoxic T cells. Radiation induced MHC-I expression on tumor cells, associated with increased susceptibility to T cell-mediated killing [91,92,93]. Similar to radiation, PDT and CAP use oxygen radicals and were shown to restore MHC-I expression in glioma and melanoma, respectively [94,95]. Additionally, PDT and radiotherapy induce MHC class I polypeptide–related sequence A (MICA) expression and upregulation of natural killer group 2D ligand (NKG2DL) on tumor cells. Both effects corresponded to increased NK cell-mediated killing of treated tumor cells [96,97]. By acting on the death receptors (e.g., Fas), the intrinsic immunogenic properties of the target cells can be altered after radiation, which consequently enhances their susceptibility to cytotoxic T cell-mediated killing [98]. The same effect was seen after treatment with BSO, inducing the formation of the CD95 death-inducing signaling complex [99]. Other chemotherapeutic regimens, which interfere with GSH, also increased the expression of death receptors [100].

ROS-inducing treatments also modulate programmed death-ligand 1 (PD-L1) expression. However, the interplay between ROS inducers and PD-L1 expression is complex, showing that both the up- and downregulation of PD-L1 expression can be induced. It is shown that different inhibitors of Trx/TrxR system decrease the PD-L1 protein level in tumor cells [101]. However, the opposite effect was reported with the TrxR inhibitor auranofin [113]. Like auranofin, arsenic trioxide induces PD-L1 expression in a dose-dependent manner in leukemic cells [127]. Besides antioxidant depletion, PD-L1 expression was increased through PI3K/Akt and STAT3 signaling in vivo and in vitro after conventionally fractionated radiotherapy [114,115]. In addition, PD-L1 expression may occur in response to tumor-targeting immune cells that release IFN-γ upon recognition of the antigen expressed by tumor cells [128]. Conversely, IFN-γ seems to represent the dominant effector molecule of the antitumor immune response after radiotherapy [93]. The same is true for different chemotherapeutics (such as doxorubicin and oxaliplatin) and other physical modalities inducing oxidative stress (including PDT and CAP), where IFN-γ was assessed as a reporter of T cell activity in response to treatments [102]. 

Many proinflammatory cytokines, including IFN-γ and tumor necrosis factor (TNF)-α, are regulated by the transcription factor NF-kB that can attract cells of the innate and adaptive immune system to mediate antitumor immune responses [129]. This highlights the paradoxical role of NF-kB, where its activation due to intermediate levels of ROS generated during lower dosages of therapeutic strategies inducing oxidative stress (e.g., radiotherapy and PDT), enhances tumor cell growth and on the other hand activates the antitumoral immunity. 

None of the described indirect effects can be generalized among all different exogenous and endogenous oxidative stress-inducing therapeutic strategies. Additionally, the effects are context and dose-dependent. Further comprehensive studies are needed to fill up the gaps in the knowledge on different ROS-inducing treatments and possible combinatorial strategies concerning their specific effect on immune response priming, recruitment of leukocytes and modification of surface molecules after treatment. 

### 2.2. Direct Effects on the Antitumoral Immune Response

#### 2.2.1. Direct Effect on Tumor-Infiltrating Immunosuppressive Cells 

Immunosuppressive cells, including tumor-associated macrophages (TAMs), regulatory T cells (Tregs) and MDSC are key components of the TME of numerous tumor types [130]. There is an interaction between tumor cells and these immune cells leading to tumor immune escape. An increased number of Tregs in tumor tissue is found in a high proportion of cancer patients and is correlated with tumor progression and poor prognosis, since Tregs help to evade host immunity. In contrast to conventional CD4+ T cells, Tregs are more resistant to oxidative stress-induced cell death [131]. This could be explained by the higher expression and secretion levels of the antioxidant molecule Trx [132]. It was shown that antioxidant Trx expression correlates with Treg representation in clinical samples of metastatic melanoma and that modulation of Trx influences the induction of Tregs and the generation of an immunotolerant cytokine profile. The addition of a Trx inhibitor decreased the number of Tregs in lung lesions. Furthermore, IFN-γ increased, whereas IL-10 and transforming growth factor (TGF)-β decreased after treatment with a Trx blocking antibody [103]. Arsenic trioxide, shown to inhibit TrxR, also induced selective depletion of Tregs and consequently increased the antitumor immune response [32,104]. Moreover, it was found that oxidative stress was the metabolic mechanism that controls tumor Treg cell functional behavior. Induction of Treg apoptosis through exogenous oxidative stress mediated the conversion of a large amount of ATP into adenosine via CD39 and CD73 and subsequently triggered an immunosuppressive cascade, tempering the therapeutic effect of immune checkpoint therapy [133]. Beyond these effects, it was shown that radiotherapy can induce TGF-β release in the TME and consequently lead to the accumulation of Tregs into the tumor tissue [116]. 

On the contrary, it was found that percentages of Tregs in the peripheral blood of cancer patients decreased significantly after radiotherapy [105]. It was confirmed by others that the reduction was mediated by the downregulation of CCL22 [106]. So far, there is no consensus concerning the effect of radiotherapy on Tregs, probably because the effect of radiotherapy on Tregs is context-dependent for different doses and tumor types. The same contradictory results are true for treatment with PDT [117]. 

Besides Tregs, certain subtypes of TAMs are also considered to have pro-tumoral functions. TAMs can differentiate from monocytes into two distinct subtypes, namely classically activated (M1) and alternatively activated (M2) macrophages with effector or suppressive function, respectively. Concerning the vulnerability of TAM to oxidative stress, M2 macrophages have lower levels of ROS compared to the M1 phenotype due to higher antioxidant activity, indicating that they will be more resistant to ROS-inducing treatments [134]. For instance, M1 macrophages were observed to be more sensitive towards radiotherapy, compared to M2 macrophages [135]. Additionally, several controversial studies have investigated the effect of chemotherapy and radiotherapy on the TAM phenotype. For example, a low dose of cyclophosphamide can promote the differentiation of M2 macrophages into M1 [107]. Similarly, low dose radiation promotes TAM skewing towards an M1 polarized phenotype and render them supportive of antitumor immunity [108]. However, higher radiation doses can polarize TAMs to an M2 phenotype promoting tumor growth, induced by factors released from irradiated cells [118]. Gold and silver nanoparticles have been shown to modulate reactive oxygen and nitrogen species production by suppressing the antioxidant system of tumor cells. When applying these nanoparticles to TAMs, there was a downregulation of TNF-α and IL-10 and an upregulation of IL-12, resulting in polarization from M2 to M1 macrophages, suggesting a radical shift from pro-tumorigenic to an anti-tumorigenic nature when TAMs undergo oxidative stress [109]. Since the polarization of TAMs is extremely dependent on the contextual signals of the TME, characterization of the oxidative stress-induced factors regulating this polarization remains to be elucidated. 

#### 2.2.2. Direct Effect on Tumor-Infiltrating Immunostimulatory Cells 

Several oxidative stress-inducing treatments have the potential to increase tumor cell immunogenicity by activating ICD and secreting immunostimulatory factors that can activate innate immune responses and elicits a tumor-specific adaptive immune response. In practice, however, the toxicity of these oxidative stress-inducing treatments to T cells, NK cells and DC limits the extent of immune stimulation and can even lead to immunosuppression [136]. Consequently, oxidative stress-inducing treatments can cause severe related lymphopenia that is associated with reduced patient survival [119].

For instance, the direct effect of radiation on lymphocytes is often immunosuppressive since most subsets of lymphocytes are radiosensitive [137]. Similar direct effects have been demonstrated after chemotherapy, PTD and CAP [119,120,121]. Nevertheless, the various lymphocyte subtypes differ in their sensitivity to exogenous induced oxidative stress. It has been demonstrated that memory and naïve T cells, as well as NK cells, are highly sensitive, whereas effector T cells, NK-T cells and Tregs are more resistant to the toxic effects of exogenous induced oxidative stress [131,138]. Additionally, the extent of immunosuppressive properties will vary with treatment schedule and dose [136,137]. In general, activated T cells and NK cells have higher antioxidant levels (GSH and Trx), necessary to buffer the rising ROS levels upon activation and proliferation of these lymphocytes, making them less vulnerable for exogenous ROS induced cell death [139,140]. For example, IL-2 activated NK cells were more resistant to H_2_O_2_-induced cell death than resting NK cells due to an upregulation of the Trx system. However, H_2_O_2_-induced cell death was also observed in these activated NK cells in the presence of a Trx inhibitor [140]. Inhibiting the antioxidant system in T cells with arsenic trioxide also induces apoptosis in T cells by enhancing oxidative stress, decreasing intracellular GSH releasing cytochrome c, activating caspases and downregulating Bcl-2 [122]. In contrast, after Trx inhibition the expression of the activation marker CD69 was significantly increased on both CD8+ T cells and NK cells [34]. 

In contrast to all lymphocyte subsets, monocytes are shown to be more resistant to exogenous ROS induced cell death [75,78,121,141]. This might be explained by a stronger antioxidant defense system in phagocytes, such as monocytes and DC, which under physiological conditions protect them against self-production of ROS during oxidative burst [142]. However, depletion of the antioxidant GSH system could also inhibit DC maturation [123]. 

In summary, ROS-inducing treatments cause direct and indirect immune effects which can be both immunostimulatory and immunosuppressive. Current research on ROS-inducing treatments mostly focuses on one immunomodulating aspect but lacks comprehensive investigation on both stimulatory and suppressive immune effects. Additionally, it is necessary to take into account the timing and location of the effects. ROS-inducing treatments can have immediate toxic and suppressive effects on tumor-infiltrating immune cells, however, can be able to attract new systemic immune cells towards the tumor, stimulating an antitumoral immune response. Therefore, it is necessary to elucidate all these challenges when investigating oxidative stress-inducing treatment modalities as a novel anticancer strategy. 

## 3. Conclusions

Preclinical studies have elucidated that an increase in ROS concentrations through exogenous and endogenous ROS-inducing therapies or a combination of both can be an efficient anticancer strategy. Hence, the influence of these treatments on the TME should be considered. Importantly, both the immunostimulatory as well as immunosuppressive effects have to be taken into account when investigating these anticancer modalities, because increasing ROS levels can be a double-edged sword with regards to immunomodulation and the effects cannot be generalized over different treatment modalities. 

## Figures and Tables

**Table 1:** Overview of immunomodulating effects of different ROS-inducing therapies.

ROS-Inducing Therapy	Effect	References
**Immunostimulating Effects**		
**Indirect Effects**		
Radiotherapy, PDT, CAP, chemotherapy (e.g., oxaliplatin, doxorubicin)	Secretion of danger signals inducing ICD (e.g., ATP, IL-1β, calreticulin, HMGB1, type I IFN)	[76,77,78,79,80,81,82,83]
Radiotherapy, PDT, CAP, chemotherapy (e.g., docetaxel, doxorubicin, oxaliplatin)	Secretion of chemokines attracting T cells (e.g., CXCL9, CXCL10, CCL5)	[83,84,85,86,87,88,89,90]
Radiotherapy, PDT, CAP, chemotherapy (e.g., topotecan)	Upregulation of MHC-I molecules on tumor cells	[91,92,93,94,95]
Radiotherapy, PDT	Upregulation of NK cell ligands (e.g., MICA, NKG2DL)	[96,97]
Radiotherapy, GSH inhibitors (e.g., BSO)	Modulation of death receptors (e.g., Fas and CD95)	[98,99,100]
Trx/TrxR inhibitors (e.g., butaselen)	Downregulation of PD-L1	[101]
Radiotherapy, PDT, CAP, chemotherapy (e.g., doxorubicin, oxaliplatin), Trx inhibitor	Secretion of proinflammatory cytokines (e.g., IFN-γ, TNF-α)	[78,93,102,103]
**Direct Effects**		
Radiotherapy, PDT, Trx/TrxR inhibitors (e.g., arsenic trioxide)	Depletion of Tregs	[103,104,105,106]
CAP, Trx inhibitor	Decrease in secretion of anti-inflammatory cytokines (e.g., IL10, TGF-β)	[78,103]
Radiotherapy, chemotherapy (e.g., cyclophosphamide), antioxidant inhibitors (e.g., noble nanoparticles)	Polarization of M2 into M1 macrophages	[107,108,109]
**Immunosuppressive Effects**		
**Indirect Effects**		
Radiotherapy, PDT, CAP, chemotherapy	Secretion of ATP modulating MDSCs	[110]
Radiotherapy	Secretion of chemokines attracting MDSCs (e.g., CXCL12)	[111,112]
Radiotherapy, Trx/TrxR inhibitors (e.g., auranofin, arsenic trioxide)	Upregulation of PD-L1	[101,113,114,115]
Radiotherapy	Secretion of anti-inflammatory cytokines (e.g., TGF-β)	[116]
**Direct Effects**		
Radiotherapy, PDT	Accumulation of Tregs	[116,117]
Radiotherapy	Polarization into M2 macrophages	[118]
Radiotherapy, PDT, CAP, chemotherapy (e.g., cisplatin, oxaliplatin), antioxidant inhibitors (e.g., arsenic trioxide)	Lymphocyte cytotoxicity	[87,116,119,120,121,122]
GSH inhibitor	Inhibition of DC maturation	[123]
